# Supplementary material for: Heat adaptation of phage T7 under an extended genetic code
Source: Virus Evol. 2021 Dec 1;7(2):veab100. doi: 10.1093/ve/veab100 (PMC8923235; doi:10.1093/ve/veab100)
Supplement: veab100_Supp [file veab100_supp.zip › Sequencing_Analysis.zip!/Sequencing_Analysis/S2/evidence/RA_29.html]

BRESEQ :: Evidence


| Read alignment evidence... | | | | | | | | | | | |
| --- | --- | --- | --- | --- | --- | --- | --- | --- | --- | --- | --- |
|  | seq id | position | | ref | new | freq | score (cons/poly) | reads | annotation | genes | product |
| \* | NC\_001604.1 | 33,279 | 0 | T | C | 33.1% | 4707.9 / inf | 4989 | intergenic (–/–) | *–/–* | –/– |
| Reads supporting (aligned to +/- strand):  **ref** base T (1621/1624);  **new** base C (787/819);  **total** (2470/2519) | | | | | | | | | | | |
| Fisher's exact test for biased strand distribution *p*-value = 5.42e-01 | | | | | | | | | | | |
| Kolmogorov-Smirnov test that lower quality scores support variant *p*-value = 9.88e-01 | | | | | | | | | | | |

Only 100 of 5016 total aligned reads displayed.  

`ATGACCTAAGGGACTTCGATATGTTCCGCATCATGCCAGCGTATGACCGCCGTGTCAATGGTGACATCGCCATCATGGGGTCTACTGGTAAAACCACTAAGGAACTTAAGGATGAGATTTTGGCTCTCAAAGCGAAAGCTGAGGGAGACGGTAAGAAGACTGGCGAGGTACATGCTTTAATGGATACCGTTAAGATTCTTACTGGTCGTGCTAGACGCAATCAGGACACTGTGTGGGAAACCTCACTGCGTGCCATCAATGACCTAGGGTTCTTCGCTAAGAACGCCTACATGGGTGCTCAGAACATTAC‑GGAGATTGCTGGGATGATTGTCACTGGTAACGTTCGTGCTCTAGGGCATGGTATCCCAATTCTG‑CGTGATACACTCTACAAGTCTAAACCAGTTTCAGCTAAGGAACTCAAGGAACTCCATGCGTCTCTGTTCGGG‑AAGGAGGTGGACCAGTTGATTCGGCCTAAACGTGCTGACATTGTGCA‑GCGCCTAAGGGAAGCAACTGATACCGGACCTGCCGTGGCGAACATCGTAGGGACCTTGAAGTATTCAACACAGGAACTGGCTGCTCGCTCTCCGTGGACTAAGCTACTGAACGGAACCACTAACTAC  >  NC_001604.1/32960‑33579`  

                                                                                                                                                                                                                                                                                                                                `|`                                                                                                                                                                                                                                                                                                                 
                       `ttCCGCATCATGCCAGCGTATGACCGCCGTGTCAATGGTGACATCGCCATCATGGGGTCTACTGGTAAAACCACTAAGGAACTTAAGGATGAGATTTTGGCTCTCAAAGCGAAAGCTGAGGGAGACGGTAAGAAGACTGGCGAGGTACATGCTTTAATGGATACCGTTAAGATTCTTACTGGTCGTGCTAGACGCAATCAGGACACTGTGTGGGAAACCTCACTGCGTGCCATCAATGACCTAGGGTTCTTCGCTAAGAACGCCTACATGGGTGCTCAGAACATTAC‑GGAGATTGCTGGga                                                                                                                                                                                                                                                                                                             <  1:496305/301‑1 (MQ=255)`  
                       `ttCCGCATCATGCCAGCGTATGACCGCCGTGTCAATGGTAACATCGCCATCATGGGGTCTACTGGTAAAACCACTAAGGAACTTAAGGATGAGATTTTGGCTCTCAAAGCGAAAGCTGAGGGAGACGGTAAGAAGACTGGCGAGGTACATGCTTTAATGGATACCGTTAAGATTCTTACTGGTCGTGCTAGACGCAATCAGGACACTGTGTGGGAAACCTCACTGCGTGCCATCAATGACCTAGGGTTCTTCGCTAAGAACGCCTACATGGGTGCTCAGAACATTAC‑GGAGATTGCTGGga                                                                                                                                                                                                                                                                                                             >  1:45217/1‑301 (MQ=255)`  
                         `ccGCATCATGCCAGCGGATGACCGCCGTGTCAATGGTGACATCGCCATCATGGGGTCTACTGGTAAAAACACTAAGGAAATTAAGGATGAGATTTTTGCTCTCAAAGCGAAAGCTGAGGGAGACGGTAAGAAGACTGGCGAGGTACATGCTTTAATGGATACCGGTAAGATTCTTACTGGTCGTGCTAGACGCAATCAGGACACTGTGTGGGAAACCTCACTGCGTGCCATCAATGACCTAGGGTTCTTCGCTAAGAACGCCTACATGGGTGCTCAGAACATTAC‑GGAGATTGCTGGgatg                                                                                                                                                                                                                                                                                                           <  1:695852/301‑1 (MQ=255)`  
                               `catGCCAGGGTATGACCGCCGTGTCAATGGTGGCATCGCCATCATGGGGTCTACTGGTAAAACCACTAAGGAACTTAAGGATGAGATTATGGCTCTAAAAGCGAAAGCTGAGGGAGACGGTAAGAAGACTGGCTAGGTACATGCTTTAATGGATACCGTTAAGGTTCTTACTGTTCGTGCTAGACGCAATCAGGACACTGTGTGGGAAACCTCACTGCGTGCCATCAATGACCTAGGGTTCTTCGCTAAGAACGCCTACATGGGTGCTCAGAACATTAC‑GGAGATTGCCGGGATGATTGTc                                                                                                                                                                                                                                                                                                     <  1:100199/301‑1 (MQ=255)`  
                                `atGCCAGCGTATGACCGCCGTGTCAATGGTGACATCGCCATCATGGGGTCTACTGGTAAAACCACTAAGGAACTTAATGATGAGATTTTGGCTCTCAAAGCGAAAGCTGAGGGAGACGGTAAGAAGACTGGCGAGGTACATGCTTTAATGGATACCGTTAAGATTCTTACTGGTCGTGCTAGACGCAATCAGGACACTGTGTGGGAAACCTCACTGCGTGCCATCAATGACCTAGGGTTCTTCGCTAAGAACGCCTACATGGGTGCTCAGAACATTAC‑GGAGATTGCCGGGATGATTGTCa                                                                                                                                                                                                                                                                                                    >  1:198055/1‑301 (MQ=255)`  
                                          `aTGACCGCCGTGTCAATGGTGACATCGCCATCATGGGGTCTACTGGTAAAACCACTAAGGAACTTAAGGATGAGATTTTGGCTCTCAAAGCGAAAGCTGAGGGAGACGGTAAGAAGACTGGCGAGGTACATGCTTTAATGGATACCGTTAAGATTCTTACTGGTCGTGCTAGACGCAATCAGGACACTGTGTGGGAAACCTCACTGCGTGCCATCAATGACCTAGGGTTCTTCGCTAAGAACGCCTACATGGGTGCTCAGAACATTAC‑GGAGATTGCTGGGATGATTgctgtctcttatac                                                                                                                                                                                                                                                                                          >  1:27122/1‑288 (MQ=255)`  
                                              `ccgccgTGTCAATGGTGACATCGCCATCATGGGGTCTACTGGTAAAACCACTAAGGAACTTAATGATGAGATTTTGGCTCTCAAAGCGAAAGCTGAGGGAGACGGTAAGAAGACTGGCGAGGTACATGCTTTAATGGATACCGTTAAGATTCTTACTGGTCGTGCTAGACGCAATCAGGACACTGTGTGGGAAACCTCACTGCGTGCCATCAATGACCTAGGGTTCTTCGCTAAGAACGCCTACATGGGTGCTCAGAACATTAC‑GGAGATTGCTGGAAAGATTGTCAcctgtctcttatac                                                                                                                                                                                                                                                                                      >  1:787020/1‑288 (MQ=255)`  
                                                  `tgtgtCAATGGTGGCATCGCCATCATGGGGTCTACTGGTAAAACCACTAAGGAACTTAAGGATGAGATTTTGGCTCTCAAAGCGAAAGCTGAGGGAGACGGTAAGAAGACTGGCGAGGTACATGCTTTAATGGATACCGTTAAGATACTTACTGGTCGTGCTAGACGCAATCAGGACACTGTGTGGGAAACCTCACTGCGTGCCATCAATGACCTAGGGTTCTTCGCTAAGAACGCCTACATGGGTGCTCAGAACATTAC‑GGAGATTGCTGGGGTGATTGTCACTGGTAACGTTCGTGctc                                                                                                                                                                                                                                                                                  <  1:488628/300‑1 (MQ=255)`  
                                                       `aaaTGGTGACATCGCCATCATGGGGTCTACTGGTAAAACCACTAAGGAACTTAAGGATGAGATTTTGGCTCTCAAAGCGAAAGCTGAGGGAGACGGTAAGAAGACTGGCGAGGTACATGCTTTAATGGATACCGTTAAGATTCTTACTGGTCGTGCTAGACGCAATCAGGACACTGTGTGGGAAACCTCACTGCGTGCCATCAATTACCTAGGGTTCTTCGCTAAGAACGCCTACATGGGTGCTCAGAACATTAC‑GGAGATTGCCGGGATGATTGTCACTGGTAACGTTCGTGCTCTAggg                                                                                                                                                                                                                                                                             <  1:512450/300‑1 (MQ=255)`  
                                                                `cATCGCCATCATGGGGTCTACTGGTAAAACCACTAAGGAACTTAAGGATGAGATTTTGGCTCTCAAAGCGAAAGCTGAGGGAGACGGTAAGAAGACTGGCGAGGTACATGCTTTAATGGATACCGTTAAGATTCTTACTGGTCGTGCTAGACGCAATCAGGACACTGTGTGGGAAACCTCACTGCGTGCCATCAATGACCTAGGGTTCTTCGCTAAGAACGCCTACATGGGTGCTCAGAACATTAC‑GGAGATTGCTGGGATGATTGTCACTGGTAACGTTCGTGCTCTAGGGCCTgtctct                                                                                                                                                                                                                                                                    >  1:528863/1‑296 (MQ=255)`  
                                                                       `atcatGGGGTCTACTGGTAAAACCACTAAGGAACTTAAGGATGAGATTTTGGCTCTCAAAGCGAAAGCTGAGGGAGACGGTAAGAAGACTGGCGAGGTACATGCTTTAATGGATACCGTTAAGATTCTTACTGGTCGTGCTAGACGCAATCAGGCCACTGTGTGGGAAACCTCACTGCGTGCCATCAATGACCTAGGGTTCTTCGCTAAGAACGCCTACATGGGTGCTCAGAACATTAC‑GGAGATTGCTGGGATGATTGTCACTGGTAACGTTCGTGCTCTAGGGCATGGTATcccctgtc                                                                                                                                                                                                                                                             >  1:780520/1‑296 (MQ=255)`  
                                                                       `atcatGGGGTCTACTGGTAAAACCACTAAGGAACTTAAGGATGAGATTTTGGCTCTCAAAGCGAAAGCTGAGGGAGACGGTAAGAAGACTGGCGAGGTACATGCTTTAATGGATACCGTTAAGATTCTTACTGGTCGTGCTAGACGCAATCAGGACACTGTGTGGGAAACCTCACTGCGTGCCATCAATGACCTAGGGTTCTTCGCTAAGAACGCCTACATGGGTGCTCAGAACATTAC‑GGAGATTGCTGGGATGATTGTCACTGGTAACGTTCGTGCTCTAGGGCATGGTATCCCAAttt                                                                                                                                                                                                                                                             >  1:419838/1‑300 (MQ=255)`  
                                                                           `tGGGGTCTACTGGTAAAACCACTAAGGAACTTAATGATGAGATTTTGGCTCTCAAAGCGAAAGCTGAGGGAGACGGTAAGAAGACTGGCGAGGTACATGCTTTAATGGATACCGTTAAGATTCTTACTGGTCGTTCTAGACGCAATCAGGACACTGTGTGGGAAACCTCACTGCGTGCCATCAATGACCTAGGGTTCTTCGCTAAGAACGCCTACATGGGTGCTCAGAACATTAC‑GGAGATTGCCGGGATGATTGTCACTGGTAACGTTCGTGCTCTAGGGCATGGTATCCCAATTCTG‑Cg                                                                                                                                                                                                                                                        >  1:160533/1‑301 (MQ=255)`  
                                                                            `ggggTCTACTGGTAAAACCACTAAGGAACTTAATGATGAGATTTTGGCTCTCAAAGCGAAAGCTGAGGGAGACGGTAAGAAGACTGGCGAGGTACATGCTTTAATGGATACCGTTAAGATTCTTACTGGTCGTGCTAGACGCAATCAGGACACTGTGTGGGAAACCTCACTGCGTGCCATCAATGACCTAGGGTTCTTCGCTAAGAACGCCTACCTTGGTGCTCAGAACATTAC‑GGAGATTGCCGGGATGATTGTCACTGGTAACGTTCGTTCTCTAGGGCATGGTATCCCAATTCTG‑CGt                                                                                                                                                                                                                                                       >  1:52142/1‑301 (MQ=255)`  
                                                                            `ggggTCTACTGGTAAAACCACTAAGGAACTTAAGGATGAGATTTTGGCTCTCAAAGCGAAAGCTGAGGGAGACGGTAAGAAGACTGGCGAGGTACATGCTTTAATGGATACCGTTAAGATTCTTACTGGTCGTGCTAGACGCAATCAGGACACTGTGTGGGAAACCTCACTGCGTGCCATCAATGACCTAGGGTTCTTCGCTAAGAACGCCTACATGGGTGCTCAGAACATTAC‑GGAGATTGCTGGGATGATTGTCGCTGGTAACGTTCGTGCTCTAGGGCATGGTATCCCAATTCTG‑CGt                                                                                                                                                                                                                                                       >  1:375811/1‑301 (MQ=255)`  
                                                                                 `cTACTGGTAAAACCACTAAGGAACTTAAGGATGAGATTTTGGCTCTCAAAGCGAAAGCTGAGGGAGACGGTAAGAAGACTGGCGAGGTACATGCTTTAATGGATACCGTTAAGATTCTTACTGGTCGTGCTAGACGCAATCATGACACTGTGTGGGAAACCTCACTGCGTGCCATCAATGACCTAGGGTTCTTCGCTAAGAACGCCTACATGGGTGCTCAGAACATTAC‑GGAGATTGCTGGGATGATTGTCACTGGTAACGTTCGTGCTCTAGGGCATGGTATCCCAATTCTG‑CGTGATac                                                                                                                                                                                                                                                  <  1:737422/301‑1 (MQ=255)`  
                                                                                   `aCTGGTAAAACCACTAAGGAACTTAAGGATGAGATTTTGGCTCTCAAAGCGAAAGCTGAGGGAGACGGTAAGAAGACTGGCGAGGTACATGCTTTAATGGATACCGTTAAGATTCTTACTGGTCGTGCTAGACGCAATCAGGACACTGTGTGGGAAACCTCACTGCGTGCCATCAATGACCTAGGGTTCTTCGCTAAGAACGCCTACATGGGTGCTCAGAACATTAC‑GGAGATTGCTGGGATGATTGTCGCTGGTAACGTTCGTGCTCTAGGGCATGGTATCCCAATTCTG‑CGTGATacac                                                                                                                                                                                                                                                <  1:444674/301‑1 (MQ=255)`  
                                                                                           `aaCCACTAAGGAACTTAAGGATGAGATTTTGGCTCTCAAAGCGAAAGCTGAGGGAGACGGTAAGAAGACTGGCGAGGTACATGCTTTAATGGATACCGTTAAGATTCTTACTGGTCGTGCTAGACGCAATCAGGACACTGTGTGGGAAACCTCACTGCGTGCCATCAATGACCTAGGGTTCTTCGCTAAGAACGCCTACATGGGTGCTCAGAACATTAC‑GGAGATTGCTGGGATGATTGTCACTGGTAACGTTCGTGCTCTAGGGCATGGTATCCCAATTCTG‑CGTGATACACTCTACAAg                                                                                                                                                                                                                                        <  1:596705/301‑1 (MQ=255)`  
                                                                                           `aaCCAATAAGGAACTTAAGGATGAGATTTTGGCTCTCAAAGCGAAAGCTGAGGGAGACGGTAAGAAGACTGGCGAGGTACATGCTTTAATGGATACCGTTAAGATTCTTACTGGTCGTGCTAGACGCAATCAGGACACTGTGTGGGAAACCTCACTGCGTGCCATCAATGACCTAGGGTTCTTCGGTAAGAACGCCTACATGGGTGCTCAGAACATTAC‑GGAGATTGCTGGGATGATTGTCACTGGTAACGTTCGTGCTCTAGGGCATGGTATCCCAATTCTG‑CGTGATACACTCTACAAg                                                                                                                                                                                                                                        <  1:91223/301‑1 (MQ=255)`  
                                                                                            `aCCACTAAGGAACTTAAGGATGAGATTTTGGCTCTCAAAGCGAAAGCTGAGGGAGACGGTAAGAAGACTGGCGAGGTACATGCTTTAATGGATACCGTTAAGATTCTTACTGGTCGTGCTAGACGCAATCAGGACACTGTGTGGGAAACCTCACTGCGTGCCATCAATGACCTAGGGTTCTTCGCTAAGAACGCCTACATGGGTGCTCAGAACATTAC‑GGAGATTGCTGGGATGATAGTCACTGGTAACGTTCGTGCTCTAGGGCATGGTATCCCAATTCTG‑CGTGATACACTCTACAAGt                                                                                                                                                                                                                                       >  1:604046/1‑301 (MQ=255)`  
                                                                                                   `aGGAACTTAAGGATGAGATTTTGGCTCTCAAAGCGAAAGCTGAGTGAGACGGTAAGAAGACTGGCGAGGTACATGCTTTAATGGATACCGTTAAGATTCTTACTGGTCGTGCTAGACGCAATCAGGACACTGTGTGGGAAACCTCACTGCGTGCCATCAATGACCTAGGGTTCTTCGCTAAGAACGCCTACATGGGTGCTCAGAACATTAC‑GGAGATTGCTGGGATGATTGTCACTGGTAACGTTCGTGCTCTAGGGCATGGTATCCCAATTCTG‑CGTGATACACTCTACAAGTCTAAAcc                                                                                                                                                                                                                                <  1:349814/301‑1 (MQ=255)`  
                                                                                                   `aGGAACTTAAGGATGAGATTTTGGCTCTCAAAGCGAAAGCTGAGGGAGACGGTAAGAAGACTGGCGAGGTACATGCTTTAATGGATACCGTTAAGATTCTTACTGGTCGTGCTAGACGCAATCAGGACACTGTGTGGGAAACCTCACTGCGTGCCATCAATGACCTAGGGTTCTTCGCTAAGAACGCCTACATGGGTGCTCAGAACATTAC‑GGAGATTGCTGGGATGCTTGTCACTGGTAACGTTCGTGCTCTAGGGCATGGTATACCAATTCTG‑CGTGATACACTCTACAAGTCTAAAcc                                                                                                                                                                                                                                <  1:300944/301‑1 (MQ=255)`  
                                                                                                     `gcaCTTAATGATGAGATTTTGGCTCTCAAAGCGAAAGCTGAGGGAGACGGTAAGAAGACTGGCGAGGTACATGCTTTAATGGATACCGTTAAGATTCTTACTGGTCGTGCTAGACGCAATCAGGACACTGTGTGGGAAACCTCACTGCGTGCCATCAATGACCTAGGGTTCTTCGCTAAGAACGCCTACATGGGTGCTCAGAACATTAC‑GGAGATTGCCGGGATGATTGTCACTGGTAACGTTCGTGCTCTATGGCATGTTATCCCAATTCTG‑CGTGATACACTCTACAAGTCTAAACCAg                                                                                                                                                                                                                              >  1:681623/3‑301 (MQ=255)`  
                                                                                                       `aaaaagagacaggaatgtggagcggaaagggaaagggGAGGGAGACGGAAAGAAGAATGGAGAGGTACAAGGTATAATGGATACCGATAAGAAACATAAGGGTCGTGCTAGAAGAAAACAGGAAACAGAGTGGGAAACCTGAATGGGTGCAATAAAGGAACAAGGGTACATGGATAAGAACGAAAAAATGGGTGCAGAGAAAATTAC‑GGAGAAAGATGGGATGAGAGAAAATGGAAAAGTTCGTGCTGTAGGGCATGGTATCCCAATTCTG‑CGTGATACACTCTACAAGTCTAAACCAGtg                                                                                                                                                                                                                            <  1:544402/276‑2 (MQ=255)`  
                                                                                                        `cTTAAGGATGAGATTTTGGCTCTCAAAGCGAAAGCTGAGGGAGACGGTAAGAAGACTGGCGAGGTACATGCTTTAATGGATACCGTTAAGATTCTTACTGGTCGTGCTAGACGCAATCAGGACACTGTGTGGGACACCTCACTGCGTGCCATCAATGACCTAGGGTTCTTCGCTAAGAACGCCTACATGGGTGCTCACAACATTAC‑GGAGATTGCCGGGATGATTGTCACTGGTAACGTTCGTGCTCTAGGGCATGGTATTCCAATTCTG‑CGTGATACACTCTACAAGTCTCAAccctgtt                                                                                                                                                                                                                           >  1:436319/1‑296 (MQ=255)`  
                                                                                                              `gATGAGATTTTGGCTCTCAAAGCGAAAGCTGAGGGAGACGGTAAGAAGACTGGCGAGGTACATGCTTTAATGGATACCGTTAAGATTCTTACTGGTCGTGCTAGACGCAATCAGGACACTGTGTGGGAAACCTCACTGCGTGCCATCAATGACCTAGGGTTCTTCGCTAAGAACGCCTACATGGGTGCTCAGAACATTAC‑GGAGATTGCTGGGATGATTGTCACTGGTAACGATCGTGCTCTAGGGCATGGTATCCCAATTCTG‑CGTGATACACTCTACAAGTCTAAACAAGTTGCAGCTa                                                                                                                                                                                                                     >  1:565137/1‑301 (MQ=255)`  
                                                                                                                   `gaTTTTGGCTCTCAAAGCGAAAGCTGAGTGAGACGGTAAGAAGACTGGCGAGGTACATGCTTTAATGGATACCGTTAAGATTCTTACTGGTCGTGCTAGACGCAATCAGGACACTGTGTGGGAAACCTCACTGCGTGCCATCAATGACCTAGGGTTCTTCGCTAAGAACGCCTACATGGGTGCTCAGAACATTAC‑GGAGATTGCTGGGATGATTGTCACTGGTAACGTTCGTGCTCTAGGGCATGGTATCCCAATTCTG‑CGTGATACACTCTACAAGTCTAAACCAGTTTCAGCTaagtaa                                                                                                                                                                                                                >  1:58104/1‑301 (MQ=255)`  
                                                                                                                   `gaTGTTGGCTCTCAAAGCGAAAGCTGAGGGAGACGGTAAGAAGACTGGCGAGGTACATGCTTTAATGGATACCGTTAAGATTCTTACTGGTCGTGCTAGACGCAATCAGGACACTGTGTGGGAAACCTCACTGCGTGCCATCAATGACCTAGGGTTCTTCGCTAAGAACGCCTACATGGGTGCTCAGAACATTAC‑GGAGATTGCCGGGATGATTGTCACTGGTAAAGTTCGTGCTTTAGGGCATGTTAGCCTAATTCTG‑AGAGATACACTATACAAGTTTAAACAAGTTTCAGCTaaggaa                                                                                                                                                                                                                >  1:221999/1‑301 (MQ=255)`  
                                                                                                                             `ctcAAAGCGAAAGCTGAGGGAGACGGTAAGAAGACTGGCGAGGTACATGCTTTAATGGATACCGTTAAGATTCTTACTGGTCGTGCTAGACGCAAACAGGACACTGTGTGGGAAACCTCACTGCGTGCCATCAATGACCTAGGGTTCTTCGCTAAGACAGCCGACATGGGTGCTCAGAACATTAC‑GGAGATTGCCGGGATGATTGTCACTGGTAACGTTCGTGCTCTAGGGCATGGTATCCCCATTCTG‑CGTGATACACTCTACAAGTCTAAACCAGTTTAAGCTaaggacctcaaggaaa                                                                                                                                                                                                      >  1:613101/1‑300 (MQ=255)`  
                                                                                                                                         `gCTGAGGGAGACGGTAAGAAGACTGGCGAGGTACATGCTTTAATGGATACCGTTAAGATTCTTACTGGTCGTGCTAGACGCAATCAGGACACTGTGTGGGAAACCTCACTGCGTGCCATCAATGACCTAGGGTTCTTCGCTAAGAACGCCTACATGGGTGCTCAGAACATTAC‑GGAGATTGCCGGGATGATTGTCACTGGTAACGTTCGTGCTCTAGGGCATGGTATCCCAATTCTG‑CGTGATACACTCTACAAGTCTAAACCAGTTTCAGCTAAGGAACTCAAGGAACTCCATGCGtctc                                                                                                                                                                                          <  1:688428/301‑1 (MQ=255)`  
                                                                                                                                                  `gaCGGTAAGAAGACTGGCGAGGTACATGCTTTAATGGATACCGTTAAGATTCTTACTGGTCGTGCTAGACGCAATCAGGACACTGTGTGGGAAACCTCACTGCGTGACATCAATGACCTAGGGTTCTTCGCTAAGAACGCCTACATGGGTGCTCAGAACATTAC‑GGAGATTGCTGGGATGATTGTCACTGGTAACGTTCGTGCTCTAGGGCATGGTATCCCAATTCTG‑CGTGATACACTCTACAAGTCTAAACCAGTTTCAGCTAAGGAACTCAAGGAACTCCATGCGTCTCTGTTCGGG‑a                                                                                                                                                                                >  1:185994/1‑301 (MQ=255)`  
                                                                                                                                                      `gTAAGAAGACTGGCGAGGTACATGCTTTAATGGATACCGTTAAGATTCTTACTGGTCGTGCTATACGCAATCAGGACACTGTGTGGGAAACCTCACTTCGTGCCATCAATGACCTATGGTTCTTCGCTAAGAACGCCTACATGGGTGCTCAGAACATTAC‑GGAGATTGCCGGGATGATTGTCACTGGTAACGTTCGTGCTCTAGGGCATGGTATCCCAATTCTG‑CGTGATACACTCTACAAGTCTAAACCAGTTTCAGCTAAGGAACACAAGGACCTCCATGCCTCCCTGTTCggtagggg                                                                                                                                                                             >  1:773199/1‑295 (MQ=255)`  
                                                                                                                                                      `gTAAGAAGACTGGCGAGGTACATGCTTTAATGGATACCGTTAAGATTCTTACTGGTCGTGCTAGACGCAATCAGGACACTGTGTGGGAAACCTCACTGCGTGCCATCAATTACCTAGGGTTCTTCGCTAAGAACGCCTACATGGGTGCTCAGAACATTAC‑GGAGATTGCTGGGATGATTGTCACTGGTAACGTTCGTGCTCTAGGGCATGGTATCCCAATTCTG‑CGTGATACACTCTACAAGTCTAAACCAGTTTCAGCTAAGGAACTCAAGGAACTCCATGCGTCTCTGTTCGGG‑Aaggg                                                                                                                                                                            >  1:29250/1‑300 (MQ=255)`  
                                                                                                                                                            `agaCTGGCGAGGTACATGCTTTAATGGATACCGTTAAGATTCTTACTGGTCGTGCTAGACGCAATCAGGACACTGTGTGGGAAACCTCACTGCGTGCCATCAATGACATAGGGTTCTTCGCTAAGAACGCCTACATGGGTGCTCAGAACATTAC‑GGAGATTGCCGGGATGATTGTCACTGGTAACGTTCGTGCTCTAGGGCATGGTATCCCAATTCTG‑CGTGATACACTCTACAAGTCTAAACCAGTTTCAGCTAAGGAACTCACGGAACTCCATGCGTCTCTGTTCGGG‑AAGGAGGTGGa                                                                                                                                                                      >  1:62182/1‑301 (MQ=255)`  
                                                                                                                                                             `agcTGGCGAGGTACATGCTTTAATGGATACCGTTAAGATTCTTACTGGTCGTGCTAGACGCAATCAGGACACTGTGTGGGAAACCTCACTGCGTGCCATCAATGACCTAGGGTTCTTCGCTAAGAACGCCTACATGGGTGCTCAGAACATTAC‑GGAGATTGCTGGGATGATTGTCACTGGTAACGTTCGTGCTCTAGGGCATGGTATCCCAATTCTG‑CGTGATACACTCTACAAGTCTAAACCAGTTTCAGCTAAGGAACTCAAGGAACTCCATGCGTCTCTGTTCGGG‑AAGGAGGTGGAc                                                                                                                                                                     <  1:249855/299‑1 (MQ=255)`  
                                                                                                                                                                `tGGCGAGGTACATGCTTTAATGGATACCGTTAAGATTCTTACTGGTCGTGCTAGACGCAATCAGGACACTGTGTGGGAAACCTCACTGCGTGCCATCAATGACCTAGGGTTCTTCGCTAAGAACGCCTACATGGGTGCTCAGAACATTAC‑GGAGATTGCTGGGATGATTATCACTGGTAACGTTCGTGCTCTAGGGCATGGTATCCCAATTCTG‑CGTGATACACTCTACAAGTCTAAACCAGTTTCAGCTAAGGAACTCAAGGAACTCCATGCGTCTCTGTTCGGG‑AAGGAGGTGGACCAg                                                                                                                                                                  <  1:823021/301‑1 (MQ=255)`  
                                                                                                                                                                      `ggTACATGCTTTAATGGATACCGTTAAGATTCTTACTGGTCGTGCTAGACGCAATCAGGACACTGTGTGGGAAACCTCACTGCGTGCCATCAATGACCTAGGGTTCTTCGCTAAGAACGCCTACATGGGTGCTCAGAACATTAC‑GGAGATTGCTGGGATGCTTGTCACTGGTAACGTTCGTGCTCTAGGGCATGGTATCCCAATTCTG‑CGTGATACACTCTACAAGTCTAAACCAGTTTCAGCTAAGGAACTCAAGGAACTCCATGCGTCTCTAATCGGG‑AAGGAGGTGGACCAGTTGAtt                                                                                                                                                            >  1:730713/1‑301 (MQ=255)`  
                                                                                                                                                                             `gCTTTAATGGATACCGTTAAGATTCTTACTGGTCGTGCTAGACGCAATCAGGACACTGTGTGGGAAACCTCACTGCGTGCCATCAATGACCTAGGGTTCTTCGCTAAGAACGCCTACATGGGTGCTCAGAACATTAC‑GGAGATTTCTGTGATGATTGTCACTGGTCACGTTCGTGCTCTAGGGCATGGTATCCCAATTCTG‑CGTGATACACTCTACAAGTCTAAACCAGTTTCAGCTAAGGAACTCAAGGAACTCCATGCGTCTCTGTTCGGG‑AAGGAGGTGGACCAGTTGATTCGGccga                                                                                                                                                     >  1:722607/1‑299 (MQ=255)`  
                                                                                                                                                                             `gCTTTAATGGATACCGTTAAGATTCTTACTGGTCGTGCTAGACGCAATCAGGACACTGTGTGGGAAACCTCACTGCGTGCCATCAATGACCTAGGGTTCTTCGCTAAGAACGCCTACATGGGTGCTCAGAACATTAC‑GGAGATTGCTGGGATGATTGTCACTGGTAACGTTCGTGCTCTAGGGCATGGTATCCCAATTCTG‑CGTGATACACTCTACAAGTCTAAACCAGTTTCAGCTAAGGAACTCAAGGAACTCCATGCGTCTCTGTTCGGG‑AAGGAGGTGGACCAGTTgctgtctctta                                                                                                                                                     >  1:366926/1‑291 (MQ=255)`  
                                                                                                                                                                              `cTTTAATGGATACCGTTAAGATTCTTACTGGTCGTGCTAGACGCAATCAGGACACTGTGTGGGAAACCTCACTGCGTGCCATCAATGACCTAGGGTTCTTCGCTAAGAACGCCTACATGGGTGCTCAGAACATTAC‑GGAGATTGCTGGGATGATTGTCACTGGTAACGTTCGTGCTCTAGGGCATGGTATCCCAATTCTG‑CGTGATACACTCTACAAGTCTAAACCAGTTTCAGCTAAGGAACTCAAGGAACTCCATGCGTCTCTGTTCGGG‑AAGGAGGTGGACCAGTTGATTCGGCCTaa                                                                                                                                                    >  1:755748/1‑301 (MQ=255)`  
                                                                                                                                                                              `cTTTAATGGATACCGTTAAGATTCTTACTGGTCGTGCTAGACGCAATCAGGACACTGTGTGGGAAACCTCACTGCGTGCCATCAATGACCTAGGGTTCTTCGCTAAGAACGCCTACATGGGTGCTCAGAACATTAC‑GGAGATTGCTGGGATGATTGTCACTGGTAACGTTCGTCCTCTAGGGCATGGTATCCCAATTCTG‑CGTGATACACTCTCCAAGTCTAAACCAGTTTCAGCTAAGGAACTCAAGGAACTCCATGCGTCTCTGTTCGGG‑AAGGAGGTGGCCCAGTTGATTCCGTCTaa                                                                                                                                                    >  1:390709/1‑301 (MQ=255)`  
                                                                                                                                                                                `ttAATGGATACCGTTAAGATTCTTACTGGTCGTGCTAGACGCAATCAGGACACTGTGTGGGAAACCTCACTGCGTGCCATCAATGACCTAGGGTTCTTCGCTAAGAACGCCTACATGGGTGCTCAGAACATTAC‑GGAGATTGCTGGGATGATTGTCGCTGGTAACGTTCGTGCTCTAGGGCATGGTATCCCAATTCTG‑CGTGATACACTCTACAAGTCTAAACCAGTATCAGCTAAGGAACTCAAGGAACTCCATGCGTCTCTGTTCGGG‑AAGGAGGTGGACCAGTTGATTCGGCCTAAAc                                                                                                                                                  <  1:342237/301‑1 (MQ=255)`  
                                                                                                                                                                                 `ataagagacaggTTAAGATTCTTACTGGTCGTGCTAGACGCAATCAGGTCACTGTGTGGGAAACCTCACTGCGTGCCATCAATGACCTAGGGTTCTTCGCTAAGAACGCCTACATGGGTGCTCAGAACATTAC‑GGGGATTGCTGGGATGATTGTCACTGGTAACGTTCATGCTCTAGGGCATGGTATCCCAATTCTG‑CGTGATACACTCTACAAGTCTAAACCAGTTTCAGCTAAGGAACTCAAGGAACTCCATGCGTCTCTGTTCGGG‑AAGGAGGTGGACCAGTTGATTCGGCCTAAACg                                                                                                                                                 <  1:83312/290‑1 (MQ=255)`  
                                                                                                                                                                                  `aaTGGATACCGTTAAGATTCTTATTGGTCGTGCTAGACGCAATCAGGACACTGTGTGGGAAACCTCACTGCGTGCCATCAATGACCTAGGGTTCTTCGCTAAGAACGCCTACATGGGTGCTCAGAACATTAC‑GGCGATTGCTGGGAGGATTGTCACTGGTAACGTTCGTTCTCTAGGGCATGGTATCCCAATTCTG‑CGTGATACACTCTACAAGTCTAAACCAGTTTCAGCTAAGGAACTCAAGGAACTCCATTCGTCTCTGTTCGGG‑AAGGCGGTGGACCAGTTGacgtctcttatcacc                                                                                                                                                >  1:764195/1‑287 (MQ=255)`  
                                                                                                                                                                                  `aaTGGATACCGTTAAGATTCTTACTGGTCGTGCTAGACGCAATCAGGACACTGTGTGGGAAACCTCACTGCGTGCCATCAATGACCTAGGGTTCTTCGCTAAGAACGCCTACATGGGTGCTCAGAACATTAC‑GGAGATTGCCGGGATGATTGTCACTGGTAACGTTCGTGCTCTAGGGCATGGTATCCCAATTCTG‑CGTGATACACTCTACAAGTCTAAACCAGTTTCAGCTATGGAACTCAAGGAACTCCATGCGTCTCTGTTCGGG‑AAGGAGGTGGACCAGTTgctgtctcttatacaa                                                                                                                                                >  1:589603/1‑286 (MQ=255)`  
                                                                                                                                                                                          `ccGTTAAGATTCTTACT‑GTCGTGCTAGACGCAATCAGGACACTGTGTGGGAAACCTCACTGCGTGCCATCAATGACCTAGGGTTCTTCGCTAAGAACGCCTACATGGGTGCTCAGAACATTAC‑GGAGATTGCCGGGATGATTGTCACTGGTAACGTTCGTGCTCTAGGGCATGGTATCCCAATTCTG‑CGTGATACACTCTACAAGTCTAAACCAGTTTCAGCTAAGGAACTCAAGGAACTCCATGCGTCTCTGTTCGGG‑AAGGAGGTGGACCAGTTGATTCGGCCTAAACGTGCTGACAtt                                                                                                                                       >  1:404021/1‑301 (MQ=255)`  
                                                                                                                                                                                            `gTTAAGATTCTTACTGGTCGTGCTAGACGCAATCAGGACACTGTGTGGGAAACCTCACTGCGTGCCATCAATGACCTAGGGTTCTTCGCTAAGAACGCCTACATGGGTGCTCAGAACATTAC‑GGAGATTGCTGGGATGATAGTCACTGGTAACGTTCGTGCTCTAGGGCATGGTATCCCAATTCTG‑CGTGATACACTCTACAAGTCTAAACCAGTTTCAGCTAAGGAACTCAAGGAACTCCATGCGTCTCTGTTCGGG‑AAGGAGGTGGACCAGTTGATTCGGCCTAAACGTGCTGACATtg                                                                                                                                      <  1:557070/301‑1 (MQ=255)`  
                                                                                                                                                                                               `atgtgtataagagacagTGCTAGACGCAATCAGGACACTGTGTGGGAAACCTCACTGCGTGCCATCAATGACCTAGGGTTCTTCGCTAAGAACGCCTACATGGGTGCTCAGAACATTAC‑GGAGATTGCTGGTATGATTGTCACTGGTAACGTTCGTGCTCTAGGGCATGGTATCCCAATTCTG‑CGTGATACACTCTACAAGTCTAAACCAGTTTCAGCTAAGGAACTCAAGGAACTCCATGCGTCTCTGTTCGGG‑AAGGAGGTGTACCAGTTGATTCGGCCTAAACGTGCTGACATTGTGc                                                                                                                                   <  1:33570/285‑1 (MQ=255)`  
                                                                                                                                                                                                     `cTTACTGGTCGTGCTAGACGCAATCAGGACACTGTGTGGGAAACCTCACTGCGTGCCATCAATGACCTAGGGTTCTTCGCTAAGAACGCCTACATGGGTGCTCAGAACATTAC‑GGAGATTGCTGGGATGATTGTCACTGGTAACGTTCGTGCTCTAGGGCATGGTATACCAATTCTG‑CGTGATACACTCTACAAGTCTAAACCAGTTTCAGCTAAGGAACTCAAGGAACTCCATGCGTCTCTGTTCGGG‑AAGGAGGTGGACCAGTTGATTCGGCCTAAACGTGCTGACATTGTGCA‑GCGcc                                                                                                                            >  1:551754/1‑301 (MQ=255)`  
                                                                                                                                                                                                     `cTTACTGGTCGTGCTAGACGCAATCAGGACACTGTGTGGGAAACCTCACTGCGTGCCATCAATGACCTAGGGTTCTTCGCTAAGAACGCCTACATGGGTGCTCAGAACATTAC‑GGAGATTGCTGGGATGATTGTCACTGGTAACGATCGTGCTCTAGGGCATGGTATCCCAATTCTG‑CGTGATACACTCTACAAGTCTAAACCAGTTTCAGCTAAGGAACTCAAGGAACTCCATGCGTCTCTGTTCGGG‑AAGGAGGTGGACCAGTTGATTCGGCCTAAACGTGCTGACAtctgtctcttat                                                                                                                             >  1:358607/1‑290 (MQ=255)`  
                                                                                                                                                                                                     `cTTACTGGTCGTGCTAGACGCAATCAGGACACTGTGGTGGAAACCTCACTGCGTGCCATCAATGACCTAGGGTTCTTCGCTAAGAACGCCTACATGGGTGCTCAGAACATTAC‑GGAGATTGCTGGGATGATTGTCGATGGCAACGGTCGTGCTCTAGGGCATGGTATCCCAATTCTG‑CGTGATACACTCTACAAGTCTAAACCAGTTTCAGCTAAGGAACTCAAGGAACTCCATGCGTCTCTGTTCGGG‑AAGGAGGTGGACCAGTTGATTCGGCCTAAACGTGCTGACATTGTGCA‑GCGcc                                                                                                                            <  1:702221/301‑1 (MQ=255)`  
                                                                                                                                                                                                         `ataagagacagaGACGCAATCAGTACACTGTGTGGGAAACCTCACTGCGTGCCATCAATGACCTAGGGTTCTTCGCTAAGAACGCCTACATGGGTGCTCAGAACATTAC‑GGAGATTGCTGGGATGATTGTCGCTGGTAACGTTCGTGCTCTAGGGCATGGTATCCCAATTCTG‑CGTGATACACTCTACAAGTCTAAACCAGTTTCAGCTAAGGAACTCAAGGAACTCCATGCGTCTCTGTTCGGG‑AAGGAGGTGGACCAGTTGATTAGGCCTAAACGTGCTGACATTGTGCA‑GCGCCTAAg                                                                                                                        <  1:137111/290‑1 (MQ=255)`  
                                                                                                                                                                                                         `atGGTCGTGCTAGACGAAATCAGGACACTGTGTGGGAAACCTCACTGCGTGCCATCAATGACCTAGGGTTCTTCACTAAGAACGCCTACATGGGTGCTCAGAACATTAC‑GGAGATTGCTGGGATGATGGTCACTGGTAACGTTCGTGCTCTAGGGCATGGTATCCCAATTCTG‑CGTGATACACTCTACAAGTCTAAACCAGTTTCAGCTAAGGAACTCAAGGAACTCCATGCGTCTCTGTTCGGG‑AAGGAGGTGGACCAGTTGATTCGGCCTAAACGTGCTGACATTGTGCA‑GCGCCTAAg                                                                                                                        <  1:816030/300‑1 (MQ=255)`  
                                                                                                                                                                                                           `ggtataagagaaagCAATCAGGACACTGTGTGGGAAACCTCACTGCGTGCCATCAATGACCTAGGGTTCTTCGCTAAGAACGCCTACATGGGTGCTCAGAACATTAC‑GGAGATTGCTGGGATGATTGTCACTGGTAACGTTCGTGCTCTAGGGCATGGTATCCCAATTCTG‑CGTGATACACTCTACAAGTCTAAACCAGTTTCAGCTAAGGAACTCAAGGAACTCCATGCGTCTCTGTTCGGG‑AAGGAGGTGGACCAGTTGATTCGGCCTAAACGTGCTGACATTGTGCA‑GCGCCTAAggg                                                                                                                      <  1:398042/288‑1 (MQ=255)`  
                                                                                                                                                                                                           `ggTCGTGCTATACGCAATTAGGACACTGAGTGGTAAACCTCACTGCGTGCCATCAATGACCTAGGGTTCTTCGCTAAGAACGCCTACATGGGTGCTCAGAACATTAC‑GTATATTGCTGGGATGATTGTCACTGGTAACGTTCATTCTCTAGGGAATGGTATCCCAATTCTG‑CGTTATACATTCTACAATTCTAAACCAGTTTCAGCTAAGGAACTCAAGGAACTCCATGCGTCTCTGTTCGGG‑AAGGAGGTGGACCAGTTGATTCGGCCTAAACGTGCTGACATTGTGCA‑GCGCCTAAggg                                                                                                                      <  1:481924/301‑1 (MQ=255)`  
                                                                                                                                                                                                           `ggTCGTGCTAGACGCAATCAGGACACTGTGTGGGAAACCTCACTGCGTGCCATCAATGGCCTAGGGTTCTTCGCTAAGAACGCCTACATGGGTGCTCAGAACATTAC‑GGAGATCGCCGGGATGATTGTCACTGGTAACGTTCGTGCTCTAGGGCATGGTATCCCAATTCTG‑CGTGATACACTCTACAAGTCTAAACCAGTTTCAGCTAAGGAACTCAAGGAACTCCATGCGTCTCTGTTCGGG‑AAGGAGGTGGACCAGTTGATTCGGCCTACACGTGCTGACATTGTGCA‑GCGCCTAAggg                                                                                                                      >  1:256479/1‑301 (MQ=255)`  
                                                                                                                                                                                                           `ggTCGTGCTAGACGCAATCAGGACACTGTGTGGGAAACCTCACTGCGTGCCATCAATGACCTAGGGTTCTTCGCTAAGAACGCCTACATGGGTGCTCAGAACATTAC‑GGAGATTGCTGGGATGATTGTCACTGGTAACGTTCGTGCTCTAGGGCATGGTATCCCAATTCTG‑CGTGATACACTCTACAAGTCTAAACCAGTTTCAGCTAAGGAACTCAAGGAACTCCATGCGTCTCTGTTCGGG‑AAGGAGGTGGACCAGTTGATTCGGCCTAAACGTGCTGACCATCTGCA‑GCGCATAAggg                                                                                                                      >  1:263146/1‑301 (MQ=255)`  
                                                                                                                                                                                                                `tGCTAGACGCAATCAGGACACTGTGTGGGAAACCTCACTGCGTGCCATCAATGACCTAGGGTTCTTCGCTAAGAACGCCTACATGGGTGCTCAGAACATTAC‑GGAGATTGCTGGGATGATTGTCACTGGTAACGTTCGTGCTCTAGGGCATGGTATCCCAATTCTG‑CGTGATACACTCTACAAGTCTAAACCAGTTTCAGCTAAGGAACTCAAGGAACTCCATGCGTCTCTGTTCGGG‑AAGGAGGTGGACCAGTTGATTCGGCCTAAACGTGCTGACATTGTGCA‑GCGCCTAAGGGAAGCa                                                                                                                 >  1:308753/1‑301 (MQ=255)`  
                                                                                                                                                                                                                    `aGACGCAATCAGGACACTGTGTGGGAAACCTCACTGCGTGCCATCAATGACCTAGGGTTCTTCGCTAAGAACGCCTACATGGGTGCTCAGAACATTAC‑GGAGATTGCTGGGATGATTGTCACTGGTAACGTTCGTGCTCTAGGGCATGGTATCCCAATTCTG‑CGTGATACACTCTACAAGTCTAAACCAGTTTCAGCTAAGGAACTCAAGGAACTCCATGCGTCTCTGTTCGGG‑AAGGAGGTGGACCAGTTGATTCGGCCTAAACGTGCTGACATTGTGCA‑GCGCCTAAGGGAAGCAACTg                                                                                                             >  1:115197/1‑301 (MQ=255)`  
                                                                                                                                                                                                                         `cAATCAGGACACTGTGTGGGAAACCTCACTGCGTGCCATCAATGACCTAGGGTTCTTCGCTAAGAACGCATACATGGGTGCTCAGAACATTAC‑GGAGATTGCTGGGATGATTGTCACTGGTAACGTTCGTGCTCTAGGGCATGGTATCCCAATTCTG‑CGTGATACACTCTACAAGTCTAAACCAGTTTCAGCTAAGGAACTCAAGGAACTCCATGCGTCTCTGTTCGGG‑AAGGAGGTGGACCAGTTGATTCGTCCTAAACGTGCTGACATTGTGCA‑GCGCCTAAGGGAAGCAACTGATAcc                                                                                                        <  1:178834/301‑1 (MQ=255)`  
                                                                                                                                                                                                                         `cAATCAGGACACTGGGTGGGAACCCGCACGGCGTGCCATCAATGACCTAGGGTTCTTCGCTAAGAACGCCTACCTGGGTGCTCAGAACATTAC‑GGAGATTGCTGGGATGATTGTCACTGGTAACGTTCGTGCTCTAGGGCATGGTATCCCAATTCTG‑CGTGATACACTCTACAAGTCTAAACCAGTTTCAGCTAAGGAACTCAAGGAACTCCATGCGTCTCTGTTCGGG‑AAGGAGGTGGACCAGTTGATTCGGCCTAAACGTGCTGACATTGTGCA‑GCGCCTAAGGGAAGCAACTGATAcc                                                                                                        <  1:808711/301‑1 (MQ=255)`  
                                                                                                                                                                                                                         `agacagggACACTGTGTGGGAAACCTCACTGCGTGCCATCAATGACCTAGGGTTCTTCGCTAAGAACGCCTACATGGGTGCTCAGAACATTAT‑GGAGATTGCTGGGATGATTGTCACTGGTAACGTTCGTGCTCTAGGGCATGGTATCCCAATTCTG‑CGTGATACACTCTACAAGTCTAAACCAGTTTCAGCTAAGGAACTCAAGGAACTCCATGCGTCTCTGTTCGGG‑AAGGAGGAGGACCAGTTGATTCGGCCTAAACGTGCTGACATTGTGCA‑GCGCCTAAGGGAAGCAACTGATAcc                                                                                                        <  1:644329/295‑1 (MQ=255)`  
                                                                                                                                                                                                                           `aTCAGGACACTGTGTGGGAAACCTCACTGCGTGCCATCAATGACCTAGGGTTCTTCGCTAAGAACGCCTACATGGGTGTTCAGAAC‑TTACAGGAGA‑TGCTGGGGAGAATTTCACTGGTCAACTGTGTGGCCTAGGGCATGGGTTTCCAATTCTGCCTTGTTCCCTTTTAAAAGCTTAACCAAGTTCCAG‑TTAGGGACTCAAAGAACTCCCTTCGTCTCTTTTCGGGAAAGGGGTTGAACAATTTGTTTCGGCTAAAACTTCCTG‑CACTTTTCAGGGGCCAAAGGGAAGC‑ACTTGTACCGGa                                                                                                     >  1:144628/1‑301 (MQ=255)`  
                                                                                                                                                                                                                           `aTCAGGACACTGTGTGGGAAACCTCACTGCGTGCCATCAATGACCTAGGGTTCTTCGCTAAGAACGCCTACATGGGTGCTCAGAACATTAC‑GGAGATTGCTGGGATGATTGTCGCTGGTAACGTTCGTGCTCTAGGGCATGGTATCCCAATTCTG‑CGTGATACACTCTACAAGTCTAAACCAGTTTCAGCTAAGGAACTCAAGGAACTCCATGCGTCTCTGTTCGGG‑AAGGAGGTGGACCAGTTGATTCGGCCTAAACGTGCTGACATTGTGCA‑GCGCCTAAGGGAAGCAACTGATACCgg                                                                                                      >  1:122428/1‑301 (MQ=255)`  
                                                                                                                                                                                                                               `ggACACTGTGTGGGAAACCTCACTGCGTGCCATCAATGACCTAGGGTTCTTCGCTAAGAACGCCTACATGGGTGCTCAGAACATTAC‑GGAGATTGCTGGGATGATTGTCACTGGTAACGTTCGTGCTCTAGGGCATGATATCCCAATTCTG‑CGTGATACACTCTACAAGTCTAAACCAGTTTCAGCTAAGGAACTCAAGGAACTCCATGCGTCTCTGTTCGGG‑AAGGAGGTGGACCAGTTGATTCGGCCTAAACGTGCGGACATTGTGCA‑GCGCCTAAGGGAAGCAACTGATACAGGACCt                                                                                                  >  1:329149/1‑301 (MQ=255)`  
                                                                                                                                                                                                                                    `cTGTGTGGGAAACCTCACTGCGTGCCATCAATGACCTATGGTTCTTCGCTAAGAACGCCTACATGGGTGCTCAGAACATTAC‑GGAGATTGCTGGGATGATTGTCACTGGTAACGTTCGTGCTCTAGGGCATGGTATCCCAATTCTT‑CGTGATACACTCTACAAGTCTAAACCAGTTTCAGCTAAGGAACTCAAGGAACTCCATGCGTCTCTGTTCGGG‑AAGGAGGTGGACCAGTTGATTCGGCCTAAACGTGCTGACATTGTGCA‑GCGCCTAAGGGAAGCAACTGATACCGGACCTGCCGt                                                                                             >  1:460205/1‑301 (MQ=255)`  
                                                                                                                                                                                                                                      `gtgtgGGAAACCTCACTGCGTGCCATCAATGACCTAGGGTTCTTCGCTAAGAACGCCTACATGGGTGCTCAGAACATTAC‑GGAGATTGCTGGGATGATTTTCACTGGTAACGTTCCTGCTCTAGGGCATGTTCTCCCAATTCTG‑CGTGATACACTCTACAAGTCTAAACCAGTTTCAGCTAAGGAACTCAAGGAACTCCATGCGTCTCTGTTCGTG‑AAGGAGGTGGACCCGTTGATTCGGCCTAAACGTGCTTACATTGTGCA‑GCGCCTAAGGGAAGCAACTGATACCGGACCTGCCGttg                                                                                           >  1:504072/1‑299 (MQ=255)`  
                                                                                                                                                                                                                                               `aCCTCACTGCGTGCCATCAATGACCTAGGGTTCTTCGCTAAGAACGCCTACATGGGTGCTCAGAACATTAC‑GGAGATTGCTGGGATGATTGTAACTGGTAACGTTCGTGCTCTAGGGCATGGTATCCCAATTCTG‑CGTGATACACTCTACATGTCTAAACCAGTTTCAGCTAAGGAACTCAAGGAACTCCATGCGTCTCTGTTCGGG‑AAGGAGGTGGACCAGTTGATTCGGCCTAAACGTTCTGACATTGTGCA‑GCGCCTAAGGGAAGCAACTGATACCGGACCTGCCGTGGCGAACATCg                                                                                  >  1:410942/1‑301 (MQ=255)`  
                                                                                                                                                                                                                                                `ccTCACTGCGTGCCATCAATGACCTAGGGTTCTTCGCTAAGAACGCCTACATGGGTGCTCAGAACATTAC‑GGAGATTGCCGGGATGATTGTCACTGGTAACGTTCGTGCTCTAGGGCATGGTATCCCAATTCTG‑CGTGATACACTCTACAAGTCTAAACCAGTTTCAGCTAAGGAACTCAAGGAACTCCATGCGTCTCTGTTCGGG‑AAGGAGGTGGACCAGTTGATTCGGCCTAAACGTGCTGACATTGTGCA‑GCGCCTAAGGGAAGCAACTGATACCGGACCTgtctcttatacacatcc                                                                                 >  1:242359/1‑285 (MQ=255)`  
                                                                                                                                                                                                                                                            `ccATCAATGACCTAGGGTTCTTCGCTAAGAACGCCTACATGGGTGCTCAGAACATTAC‑GGAGATTGCTGGGATGATTGTCACTGGTAACGTTCGTGCTCTAGGGCATGGTATCCCAATTCTG‑CGTGATACACTCTACAAGTCTAAACCAGTTTCAGCTAAGGAACTCAAGGAACTCCATGCGTCTCTGTTCGGG‑AAGGAGGTGGACCAGTTGATTCGGCCTAAACGTGCTGACATTGTGCA‑GCGCCTAAGGTAAGCAACTGATACCGGACCTGCCGTGGCGAACATCGTAGGGACCTTGaa                                                                     >  1:212777/1‑301 (MQ=255)`  
                                                                                                                                                                                                                                                             `gagacaggACCTAGGGTTCTTCGCTAAGAACGCCTACATGGGTGCTCAGAACATTAC‑GGAGATTGCGGGGATGATTGTCACTGGTAACGTTCGTGCTCTAGGGCATGGTATCCCAATTCTG‑CGTGATACACTCTACAAGTCTAAACCAGTTTCAGCTAAGGAACTCAAGGAACTCCATGCGTCTCTGTTCAGG‑AAGGAGGTGGACCAGTTGATTCGGCCTAAACGTGCTGACATTGTGCA‑GCGCCTAAGGGAAGCAACTGATACCGGACCTGCCGTGGCGAACATCGTAGGGACCTTGAAg                                                                    <  1:285127/294‑1 (MQ=255)`  
                                                                                                                                                                                                                                                             `gaTCAATGACCTAGGGTTCTTCGCTAAGAACGCCTACATGGGTGCTCAGAACATTAC‑GGAGATTGCTGGGATGATTGTCACTGGTAACGTTCGTGCTCTAGGGCATGGTATCCCAATTCTG‑CGTGATACACTCTACAAGTCTAAACCAGTTTCAGCTAAGGAACTCAAGGAACTCCATGCGTCTCTGTTCGGG‑AAGGAGGTGGACCAGTTGATTCGGCCTAAACGTGCTGACATTGTGCA‑GCGCCTAAGGGAAGCAACTGATACCGGACCTGCCGTGGCGAACATCGTAGGGACCTTGAAg                                                                    <  1:635367/300‑1 (MQ=255)`  
                                                                                                                                                                                                                                                             `cATCAATGACCTAGGGTTCTTCGCTAAGAACGCCTACATGGGTGCTCAGAACATTAC‑GGAGATTGCCGGGATGATTGTCACTGGTAACGTTCGTGCTCTAGGGCATGGTATCCCAATTCTG‑CGTGATACACTCTACAAGTCTAAACCAGTTTCAGCTAAGGAACTCAAGGAACTCCATGCGTCTCTGTTCGGG‑AAGGAGGTGGACCAGTTGATTCGGCCTAAACGTGCTGACATTGTGCA‑GCGCCTAAGTGAAGCAACTGATACCGGACCTGCCGTGGCGAACATCGTAGGGACCTTGAAg                                                                    <  1:169771/301‑1 (MQ=255)`  
                                                                                                                                                                                                                                                             `cATCAATGACCTAGGGTTCTTAGCTAAGAACGCCTACATGGGTGCTCAGAACATTAC‑GGAGATTGCTGGGATGATTGTCACGGGTAACGTTCGTGCTCTAGGGCATGGTATCACAATTCTG‑CGTGATACACTCTACAAGTCTAAACCAGTTTCAGCTAAGGAACTCAAGGAACTCCATGCGTCTCTGTTCGGG‑AAGGAGGTGGACCAGTTGATTCGGCCTAAACGTGCTGACATTGTGCA‑GCGCCTAAGGGAAGCAACTGATACCGGACCTGCCGTGGCGAACATCGTAGGGACCTTGAAg                                                                    <  1:750128/301‑1 (MQ=255)`  
                                                                                                                                                                                                                                                                  `aTGACGTAGGGTTCTTCGCTAAGAACGCCTACATGGGTGCTCAGAACATTAC‑GGAGATTGCTGGGATGATTGTCACTGGTAACGTTCGTGCTCTAGGGCATGGTATCCCAATTCTG‑CGTGATACACTCTACAAGTCTAAACCAGTTGCAGCTAAGGAACTCAAGGAACTCCATGCGTCTCTGTTCGGG‑AAGGAGGTGGACCAGTTGATTCGGCCTAAACGTGCTGACATTGTGCA‑GCGCCTAAGGGAAGCAACTGATACCGGACCTGCCGTGGCGAACATCGTAGGGACCTTGAAGTATTc                                                               <  1:4671/301‑1 (MQ=255)`  
                                                                                                                                                                                                                                                                     `aCCTAGGGTTCTTCGCTAAGAACGCCTACATGTGTGCTCAGAACATTAC‑GGAGATTGCCGGGATGATTGTCACTGGTAACGTTCGTGCTCTAGGGCATGGTATCCCAATTCTT‑CGTGATACACTCTACAAGTCTAAACCAGTTTCAGCTAAGGAACTCAAGGAACTCCATGCGTCTCTGTTCGGG‑AAGGAGGTGGACCAGTTGATTCGGCCTAAACGTGCTGACATTGTGCA‑GCGCCTAAGGGAAGCAACTGATACCGGACCTGCCGTGGCGAACATCGTAGGGACCTTGAAGTATTCAac                                                            <  1:205338/301‑1 (MQ=255)`  
                                                                                                                                                                                                                                                                     `aCCTAGGGTTCTTCGCTAAGAACGCCTACATGGGTGCTCAGAACATTAC‑GGAGATTGCTGGGATGATTGTCACTGGTAACGTTCGTGCTCTAGGGCATGGTATCCCAATTCTG‑CGTGATACACTCTACAAGTCTAAACCAGTTTCAGCTAAGGAACTCAAGTAACTCCATGCGTCTCTGTTCGGG‑AAGGAGGTGGACCAGTTGATTCTGCCTAAACGTGCTGACATTGTGCA‑GCGCCTAAGGGAAGCAACTGATACCGGACCTGCCGTGGCGGACATCGTAGGGACCTTGAAGTATTCAac                                                            >  1:127978/1‑301 (MQ=255)`  
                                                                                                                                                                                                                                                                      `gcTAGGTTTCTTCGCTAAGAACGCCTACATGGGTGCTCAGAACATTAC‑GGAGATTGCT‑GGATGATTGTCACTGGTAACGTTCGTGCTCTAGGGCATGGTATCCCAATTCTG‑CGTGATACACTCTACAAGTCTAAACCAGTTTCAGCTAAGGAACTCAAGGAACTCCATGCGTCTCTGTTCGGG‑AAGGAGGTGGACCAGTTGATTCGGCCTAAACGTGCTGACATTGTGCA‑GCGCCTAAGGGAAGCAACTGATACCGGACCTGCCGTGGCGAACATCGTAGGGACCTTGAAGTATTCAacac                                                          <  1:383106/300‑1 (MQ=255)`  
                                                                                                                                                                                                                                                                       `cTAGGGTTCTTCGCTAAGAACGCCTACATGGGTGCTCAGAACATTAC‑GGAGATTGCTGGGATGATTGTCACTGGTAACGTTCGTGCTCTAGGGCATGGTATCCCAATTCTG‑CGTGATACACACTACAAGTCTAAACCAGTTTCAGCTAAGGAACTCAAGGAACTCCATGCGTCTCTGTTCGGG‑AAGGAGGTGGACCAGTTGATTCGGCCTAAACGTGCTGACATTGTGCA‑GCGCCTAAGGGAAGCAACTGATACCGGACCTGCCGTGGCGAACATCGTAGGGACCTTGAAGTATTCAacac                                                          <  1:229159/301‑1 (MQ=255)`  
                                                                                                                                                                                                                                                                                 `tcGCTAAGAACGCCTACATGGGTGCTCAGAACATTAC‑GGAGATTGCTGGGATGATTGTCACTGGTAACGTTCGTGCTCTAGGGCATGGTATCCCAATTCTG‑CGTGATACACTCTACAAGTCTAAACCAGTTTCAGCTAAGGAACTCAAGGAACTCCATGCGTCTCTGTTCGGG‑AAGGAGGTGGACCAGTTGATTCGGCCTAAACGTGCTGACATTGTGCA‑GCGCCTAAGGGAAGCAACTGATACCGGACCTGCCGTGGCGAACATCGTAGGGACCTTGAAGTATTCAACACAGGAACTGgc                                                <  1:537450/301‑1 (MQ=255)`  
                                                                                                                                                                                                                                                                                   `gCTAAGAACGCCTACATGGGTGCTCAGAACATTAC‑GGAGATTGCTGGGATGATTGTCACTGGTAACGTTCGTGCTCTAGGGCATGGTATCCCAATTCTG‑CGTGATACACTCTACAAGTCTAAACCAGTTTCAGCTAAGGAACTCAAGGAACTCCATGCGTCTCTGTTCGGG‑AAGGAGGTGGACCAGTTGATTCGGCCTAAACGTGCTGACATTGTGCA‑GCGCCTAAGGGAAGCAACTGATACCGGACCTGCCATGGCGAACATCGTAGGGACCTTGAAGTATTCAACACAGGAACTGgctg                                              >  1:321867/1‑301 (MQ=255)`  
                                                                                                                                                                                                                                                                                      `aaGAACGCCTACATGGGTGCTCAGAACATTAC‑GGAGATTGCTGGGATGATTGTCACTGGTAACGTTCGTGCTCTAGGGCATGGTATCCCGATTCTG‑CGTGATACACTCTACAAGTCTAAACCAGTTTCAGCTAAGGAACTCAAGGAACTCCATGCGTCTCTGTTCGGG‑AAGGAGGTGGACCAGTTGATTCGGCCTAAACGTGCTGACATTGTGCA‑GCGCCTAAGGGAAGCAACTGATACCGGACCTGCCGTGGCGAACATCGTAGGGACCTTGAAGTATTCAACACAGGAACTGGCTgctc                                           >  1:31432/1‑301 (MQ=255)`  
                                                                                                                                                                                                                                                                                        `gAACGCCTACATGGGTGCTCAGAACATTAC‑GGAGATTGCCGGGATGATTGTCACTGGTAACGTTCGTGCTCTAGGGCATGGTATCCCAATTCTG‑CGTGATACACTCTACAAGTCTAAACCAGTTTCAGCTAAGGAACTCAAGGAACTCCATGCGTCTCTGTTCGGG‑AAGGAGGTGGACCAGTTGATTCGGCCTAAACGTGCTGACATTGTGCA‑GCGCCTAAGGGAAGCAACTGATACCGGACCTGCCGTGTCGAACATCGTAGGGACCTTGAAGTATTCAACACAGGAACTGGCTgctcgc                                         >  1:474684/1‑301 (MQ=255)`  
                                                                                                                                                                                                                                                                                           `cGCCTACATGGGTGCTCAGAACATTAC‑GGAGATTGCTGGGATGATTGTCGCTGGTAACGTTCGTGCTCTAGGGCATGGTATCCCAATTCTG‑CGTGATACACTCTACAAGTCTAAACCAGTTTCAGCTAAGGAACTCAAGGAACTCCATGCGTCTCTGTTCGGG‑AAGGAGGTGGACCAGTTGATTCGGCCTAAACGTGCTGACATTGTGCA‑GCGCCTAAGGGAAGCAACTGATACCGGACCTGCCTTGGCGAACATCGTAGGGACCTTGAAGTATTCCACACAGGAACTGGCTGCTCGctct                                      >  1:106520/1‑301 (MQ=255)`  
                                                                                                                                                                                                                                                                                           `cGCCTACATGGGTGCTCAGAACATTAC‑GGAGATTGCCGGGATTATTGTCACTGGTAACGTTCGTGCTCTAGGGCATGGTATCCCAATTCTG‑CGTGATACACTCTACAAGTCTAAACCAGTTTCAGCTAAGGAACTCAAGGAACTCCATGCGTCTCTGTTCGGG‑AAGGAGGTGGACCAGTTGATTCGGCCTAAACGTGCTGACATTGTGCA‑GCGCCTAAGGGAAGCAACTGATACCGGATCTGCCGTGGCGAACATCGTAGGGACCTTGAAGTATTCAACACAGGAACTGGCTGCTCGctct                                      >  1:708649/1‑301 (MQ=255)`  
                                                                                                                                                                                                                                                                                                 `cATGGGTGCTCAGAACATTAC‑GGAGATTGCCGGGATGATTGTCACTGGTAACGTTCGTGCTCTAGGGCATGGTATCCCAATTCTG‑CGTGATACACTCTACAAGTCTAAACCAGTTTCAGCTAAGGAACTCAAGGAACTCCATGCGTCTCTGTTCGGG‑AAGGAGGTGGACCAGTTGATTCGGCCTAAACGTGCTGACATTGTGCA‑GCGCCTAAGGGAAGCAACTGATACCGGACCTGCCGTGGCGAACATCGTAGGGACCTTGAAGTATTCAACACAGGAACTGGCTGCTCGCTCTCCgctg                                >  1:674179/1‑298 (MQ=255)`  
                                                                                                                                                                                                                                                                                                 `cATGGGTGCTCAGAACATTAC‑GGAGATTGCCGGGATGATTGTCACTGGTAACGTTCGTGCTCTAGGGCATGGTATCCCAATTCTG‑CGTGATACACTCTACAAGTCTAAACCAGTTTCAGCTAAGGAACTCAAGGAACTCCATGCGTCTCTGTTCGGG‑AAGGAGGTGGACCAGTTGATTCGGCCTAAACGTGCTGACATTGTGCA‑GCGCCTAAGGGAAGCAACTGATACCGGACCTGCCGTGGCGAACATCGTAGGGACCTTGAAGTATTCAACACAGGAACTGGCTGCTCGCTCTCCGTgg                                >  1:628328/1‑301 (MQ=255)`  
                                                                                                                                                                                                                                                                                                  `aTGGGTGCTCAGAACATTAC‑GGAGATTGCTGGGATGATTGTCACTGGTAACGTTCGTGCTCTAGGGCATGGTATCCCAATTCTG‑CGTGATACACTCTACAAGTCTAAACCAGTTTCAGCTAAGGAACTCAAGGAACTCCATGCGTCTCTGTTCGGG‑AAGGAGGTGGACCAGTTGATTCGGCCTAAACGTGCTGACATTGTGCA‑GCGCCTAAGGGAAGCAACTGATACCGGACCTGCCGTGGCGAACATCGTAGGGACCTTGAAGTATTCAACACAGGAACTGGATGCTCGCTCTCCGTGGa                               <  1:652216/301‑1 (MQ=255)`  
                                                                                                                                                                                                                                                                                                   `tGGGTGCTCAGAACATTAC‑GGAGATTGCTGGGATGATTGTCACTGGTAACGTTCGTGCTCTAGGGCATGGTATCCCAATTCTG‑CGTGATACACTCTACAAGTCTAAACCAGTTTCAGCTAAGGAACTCAAGGAACTCCATGCGTCTATGTTCGGG‑AAGGAGGTGGACCAGTTGATTCGGCCTAAACGTGCTGACATTGTGCA‑GCGCCTAAGGGAAGCAACTGATACCGGACCTGCCGTGGCGAACATCGTAGGGACCTTGAAGTATTCAACACAGGAACTGGCTGCTCGCTCTCCGTGGAc                              <  1:277932/301‑1 (MQ=255)`  
                                                                                                                                                                                                                                                                                                    `gggTGCTCAGAACATTAC‑GGAGATTGCTGGGATGATTGTCACTGGTAACGTTCGTGCTCTAGGGCATGGTATCCCAATTCTG‑CGTGATACACTCTACAAGTCTAAACCAGTTTCAGCTAAGGAACTCAAGGAACTCCATGCGTCTCTGTTCGGG‑AAGGAGGTGGACCAGTTGATTCGGCCTAAACGTGCTGACATTGTGCA‑GCGCCTAAGGGAAGCAACTGATACCGGACCTGCCGTGGCGAACATCGTAGGGACCTTGAAGTATTCAACACAGGAACTGGCTGCTCGCTCTCCGTGGACt                             <  1:667096/301‑1 (MQ=255)`  
                                                                                                                                                                                                                                                                                                      `gTGCTCAGAACATTAC‑GGAGATTGCCGGGATGATTGTCACTGGTAACGTTCGTGCTCTAGGGCATGGTATCCCAATTCTG‑CGTGATACACTCTACAAGTCTAATCCAGTTTCAGCTAAGGAACTCAAGGAACTCCATGCGTCTCTGTTCGGG‑AAGGAGGTGGACCAGTTGATTCGGCCTAAACGTGCTGACATTGTGCA‑GCGCCTAAGGGAAGCAACTGATACCGGACCTGCCGTGGCGAACATCGTAGGGACCTTGAAGTATTCAACACAGGAACTGGCTGCTCGCTCTCCGTGGACTaa                           >  1:794248/1‑301 (MQ=255)`  
                                                                                                                                                                                                                                                                                                         `cTCAGAACATTAC‑GGAGATTGCTGGGATGATTGTAACTGGTAACGTTCGTGCTCTAGGGCATGGTATCCCAATTCTG‑CGTGATACACTCTACAAGTCTAAACCAGTTTCAGCTAAGGAACTCAAGGAACTCCATGCGTCTCTGTTCGGG‑AAGGAGGTGGACCAGTTGATTCGGCCTAAACGTGCTGACATTGTGCA‑GCGCCTAAGGGTAGCAACTGATACCGGACCTGCCGTGGCGAACATCGTAGGGACCTTGAAGTATTCAACACAGGAACTGGCTGCTCGCTCTCCGTGGACTAAGCt                        <  1:429400/301‑1 (MQ=255)`  
                                                                                                                                                                                                                                                                                                             `gAACATTAC‑GGAGATTGCTGGGATGATTGTCACTGGTAACGTTCGTGCTCTAGGGCATGGTATCCCAATTCTG‑CGTGATACACTCTACAAGTCTAAACCAGTTTCAGCTAAGGAACTCAAGGAACTCCATGCGTCTCTGTTCGGG‑AAGGAGGTGGACCAGTTGATTCGGCCTAAACGTGCTGACATTGTGCA‑GCGCCTAAGGGAAGCAACTGATACCGGACCTGCCGTGGCGAACATCGTAGGGACCTTGAAGTATTCAACACAGGAACTGGCTGCTCGCTCTCCGTGGACTAAGCTACTg                    >  1:66004/1‑301 (MQ=255)`  
                                                                                                                                                                                                                                                                                                             `gAACATTAC‑GGAGATTGCTGGGATGATTGTCACTGGTAACGTTCGTGCTCTAGGGCATGGTATCCCAATTCTG‑CGTGATACACTCTACAAGTCTAAACCAGTTTCAGCTAAGGAACTCAAGGAACTCCATGCGTCTCTGTTCGGG‑AAGGAGGTGGACCAGTTGATTCGGCCTAAACGTGCTGACATTGTGCA‑GCGCCTAAGGGAAGCAACTGATACCGGACCTGCCGTGGCGAACATCGTAGGGACCTTGAAGTATTCAACACAGGAACTGGCTGCTCGCTCTCCGTGGACTAAGCTACTg                    >  1:191882/1‑301 (MQ=255)`  
                                                                                                                                                                                                                                                                                                               `aCATTAC‑GGAGATTGCTGGGATGATGGTCACTGGTAACGTTCGTGCTCTAGGGCATGGTATCCCAATTCTG‑CGTGATACACTCTACAAGTCTAAACCAGTTTCAGCTAAGGAACTCAAGGAACTCCATGCGTCTCTGTTCGGG‑AAGGAGGTGGACCAGTTGATTCGGCCTAAACGTGCTGACATTGTGCA‑GCGCCTAAGGGAAGCACCTGATACCGGACCTGCCGTGGCGAACATCGTAGGGACCTTGAAGTATTCAACACAGGAACTGGCTCCTCGCTCTCCGTGGACTAAGCTACTGaa                  >  1:715952/1‑301 (MQ=255)`  
                                                                                                                                                                                                                                                                                                                  `ttAC‑GGAGATTGCTGTGATGATTGTCACTGGTAACGTTCGTGCTCTAGCGCATGGTATCCCAATTCTG‑CGTGATACACTCTACAAGTCTAAACCAGTTTCAGCTAAGGAACTCAAGGAACTCCACGCGTCTCTGTTCGGG‑AAGGAGGTGGACCAGTTGATTCGGCCTAAACGTGCTGACATTGTGCA‑GCGCCTAAGGGAAGCAACTGATACCGGACCTGCCGTGGCGAACATCGTAGGGACCTTGAAGTATTCAACACAGGAACTGGCTGCTCGCTCTCCGTGGACTAAGCTACTGAACgg               <  1:237193/301‑1 (MQ=255)`  
                                                                                                                                                                                                                                                                                                                  `ttAC‑GGAGATTGCTGGGATGATTGTCACTGGTAACGTTCGTGCTCTAGGGAATGGTATCCCAATTCTG‑CGTGATACACTCTACCAGTCTAAACCAGTTTCAGCTAAGGAACTCAAGGAACGCCACGCGTCTCTGTTCGGG‑AAGGAGGTGGACCAGTTGATTCGGCCTAAGCGTGCTGACATTGTGCA‑GCGCCTAAGGGAAGCAACTGATACCGGACCTGCGGTGGCGAACATCGTAGGGACCTTGAAGTATTCAACACAGGAACTGGCTGCTCGCTCTCCGTGGACTAAGCTACTGAACgg               <  1:153973/301‑1 (MQ=255)`  
                                                                                                                                                                                                                                                                                                                           `aTTGCTGGGATGATTGTCGCTAGGAACGTTCGTGCTCAAGGGCAAGGTATCCCAAGACTG‑CGTGAAACAATCTACAAGTCTAAACCAGTGTCAGCTAAAGAACTCAAGGAACTCCATGCGTCTCTGTTCGGG‑AAGGAGGTGGACCAGTTGATGCGGCCTAAACGTGCTGACATTGTGCA‑GCGCCTAAGGGAAGCAACTGATACCGAACCTGCCGTGGCGAACATCGTAGAGACCTTGAAATATTCAACACAGGAACTGGCTGCTCGCTCTCCGTGGACTAAGCTACTGAACGGAACCacta       <  1:742036/301‑1 (MQ=255)`  
                                                                                                                                                                                                                                                                                                                            `ttGCTGGGATGATGGTCACTGGTAACGTTCGTGCTCTAGGGCATGGTATCCCAATTCTG‑CGTGATACACTCTACAAGTCTAAACCAGTTTCAGCTAAGGAACTCAAGGAACTCCATGCGTCTCTGTTCGGG‑AAGGAGGTGGACCAGTTGATTCGGCCTAAACGTGCTGACATTGTGCA‑GCGCCTAAGGGAAGCAACTGATACCGGACCTGCCGTGGCGAACATCGTAGGGACCTTGAAGTATTCAACACAGGAACTGGCTGCTCGCTCTCCGTGGACTAAGCTACTGAACGGAACCactaa      >  1:574223/1‑301 (MQ=255)`  
                                                                                                                                                                                                                                                                                                                            `ttGCCGGGATGATTGTCACTGGTAACGTTCGTGCTCTAGGGCATGGTATCCCAATTCTG‑CGTGATACACTCTACAAGTCTAAACCAGTTTCAGCTAAGGAACTCAAGGAACTCCATGCGTCTCTGTTCGGG‑AAGGAGGTGGACCAGTTGATTCGGCCTAAACGTGCTGACATTGTGCA‑GCGCCTAAGGGAAGCAACTGATACCGGACCTGCCGTGGCGAACATCGTAGGGACCTTGAAGTATTCAACACAGGAACTGGCTGCTCGCTCTCCGTGGACTAAGCTACTGAACGGAACCactaa      >  1:802306/1‑301 (MQ=255)`  

                                                                                                                                                                                                                                                                                                                                `|`                                                                                                                                                                                                                                                                                                                 
`ATGACCTAAGGGACTTCGATATGTTCCGCATCATGCCAGCGTATGACCGCCGTGTCAATGGTGACATCGCCATCATGGGGTCTACTGGTAAAACCACTAAGGAACTTAAGGATGAGATTTTGGCTCTCAAAGCGAAAGCTGAGGGAGACGGTAAGAAGACTGGCGAGGTACATGCTTTAATGGATACCGTTAAGATTCTTACTGGTCGTGCTAGACGCAATCAGGACACTGTGTGGGAAACCTCACTGCGTGCCATCAATGACCTAGGGTTCTTCGCTAAGAACGCCTACATGGGTGCTCAGAACATTAC‑GGAGATTGCTGGGATGATTGTCACTGGTAACGTTCGTGCTCTAGGGCATGGTATCCCAATTCTG‑CGTGATACACTCTACAAGTCTAAACCAGTTTCAGCTAAGGAACTCAAGGAACTCCATGCGTCTCTGTTCGGG‑AAGGAGGTGGACCAGTTGATTCGGCCTAAACGTGCTGACATTGTGCA‑GCGCCTAAGGGAAGCAACTGATACCGGACCTGCCGTGGCGAACATCGTAGGGACCTTGAAGTATTCAACACAGGAACTGGCTGCTCGCTCTCCGTGGACTAAGCTACTGAACGGAACCACTAACTAC  >  NC_001604.1/32960‑33579`

  

Alignment Legend

Aligned base mismatch/match (shaded by quality score): `ATCG/ATCG` `< 3 ≤` `ATCG/ATCG` `< 11 ≤` `ATCG/ATCG` `< 25 ≤` `ATCG/ATCG` `< 36 ≤` `ATCG/ATCG` `< 38 ≤` `ATCG/ATCG`

Unaligned base: `atcg`    Masked matching base: `atcg`    Alignment gap: `‑`    Deleted base: `‑`
